# Plastid DNA Diversity Is Higher in the Island Endemic Guadalupe Cypress than in the Continental Tecate Cypress

**DOI:** 10.1371/journal.pone.0016133

**Published:** 2011-01-20

**Authors:** Patricia Rosas Escobar, David S. Gernandt, Daniel Piñero, Pedro P. Garcillán

**Affiliations:** 1 Facultad de Ciencias, Universidad Autónoma de Baja California, Ensenada, Baja California, Mexico; 2 Departamento de Zoología, Instituto de Biología, Universidad Nacional Autónoma de México, Distrito Federal, Mexico; 3 Departamento de Botánica, Instituto de Biología, Universidad Nacional Autónoma de México, Mexico, Distrito Federal, Mexico; 4 Departamento de Ecología Evolutiva, Instituto de Ecologia, Universidad Nacional Autónoma de México, Distrito Federal, Mexico; 5 Centro de Investigaciones Biológicas del Noroeste, La Paz, Baja California Sur, Mexico; University of California Riverside, United States of America

## Abstract

**Background:**

*Callitropsis guadalupensis* (Guadalupe cypress) is endemic to Guadalupe Island, Mexico, where it is the dominant species of the only forest. The species has suffered declining numbers following the introduction of goats to the island over 150 years ago. *Callitropsis guadalupensis* is closely related to *Callitropsis forbesii* (Tecate cypress), distributed in small isolated populations in mainland Baja California and southern California. The objective of the present study was to compare the genetic diversity of the island endemic to the continental species.

**Methodology/Principal Findings:**

We measured genetic diversity in *Callitropsis guadalupensis* (n = 54) from Guadalupe Island and in *Callitropsis forbesii* (n = 100) from five populations in mainland Baja California. The plastid DNA *trnS*-*trnG* spacer and the *trnL-trnF* region were chosen for characterization. Thirty-four haplotypes were observed, of which six were shared between both species. One of these haplotypes was also shared with three other species, *Callitropsis lusitanica*, *Callitropsis montana*, and *Callitropsis stephensonii*. Haplotype diversity (*h*) and nucleotide diversity (*π*) were significantly higher for *Callitropsis guadalupensis* (*h* = 0.698, *π = *0.00071) than for *Callitropsis forbesii* (*h* = 0.337, *π = *0.00024).

**Conclusions/Significance:**

*Callitropsis guadalupensis* shows no evidence of a founder effect or of a genetic bottleneck, and can be added to a growing list of insular species with higher genetic diversity than their mainland relatives.

## Introduction

The importance of genetic diversity within and between populations lies in the possibility that the variation confers a capacity to respond to environmental changes, including climate change, pollution, introduced species, disease, pathogens, or parasites [1]. Most comparisons of genetic variation between closely related island and continental populations find reduced genetic variation and elevated extinction rates in island populations; these tendencies have been attributed to founder effects, low average population size, and inbreeding [2]. Islands off the west coast of southern California, U.S.A. and the Baja California peninsula, Mexico, including the Channel Islands, Guadalupe Island, and Cedros Island, are floristically similar to the mainland [3] and as such are ideal for comparing the variables that affect genetic diversity. Guadalupe Island, located 260 km west of the peninsula of Baja California, is unique in having been formed by two shield volcanoes approximately 7 million years ago [4], and presumably in never having been in contact with the mainland [5]. Its isolation relative to other islands off of California may have resulted in a stronger founder effect and hence lower genetic diversity and higher extinction risk for its flora and for less vagile components of its fauna.

**Figure 1:**
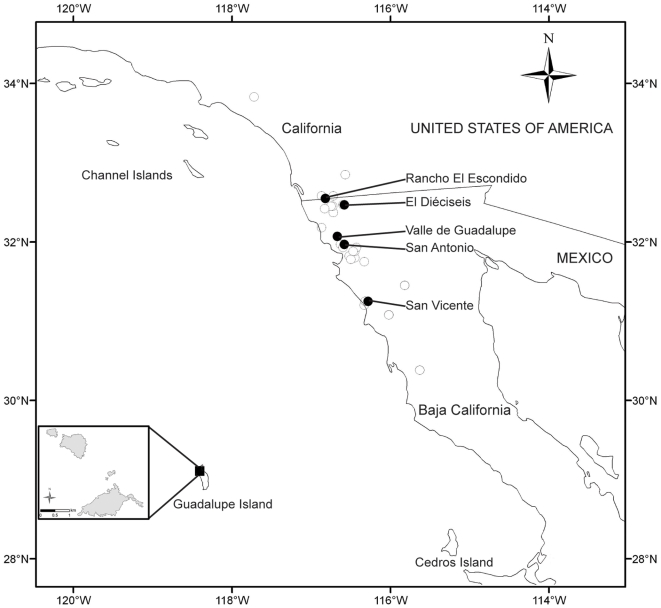
The geographic distribution of *Callitropsis guadalupensis* and *C. forbesii*. The circles in the northwest of Baja California and southern California represent the known populations of *C. forbesii*; dark circles represent sampled populations. The square on Guadalupe Island represents the location of the only population of *C. guadalupensis*. (Inset) The spatial distribution of the *C. guadalupensis* forest on Guadalupe Island.

*Callitropsis guadalupensis* (S. Watson) D. P. Little (Guadalupe cypress) is endemic to Guadalupe Island, where it forms four forest patches of 8.8, 46.9, 4.3, and 87.7 ha at the north end of the island at an elevation of 950–1300 m (Figure 1). The presence of *C. guadalupensis* forest in this zone of the island likely is due to a greater availability of water from precipitation and cloud condensation [5], [6]. In 2005, the population size of *C. guadalupensis* was estimated at 14,700 individuals [7]. The forest has been reduced in size by feral goats (*Capra hircus*) that were introduced to the island over 150 ago and have decimated the native flora [5]. The goats altered the population dynamics of *C. guadalupensis*, killing mature trees by eating their bark, and impeding new recruitment by browsing seedlings [5]. This decline of its only population is the principal reason that *C. guadalupensis* is currently classified as endangered by the Mexican government [8] and is included in the IUCN Red List [9], [10]. Since 2004 when a multi-institutional governmental program was implemented to eradicate the goats from the island, population recruitment of the cypress population was observed for the first time, as was a notable recovery of other vegetation [7], [11]. Nevertheless, threats to the Guadalupe cypress persist; in September of 2008 a fire burned part of the forest.

*Callitropsis forbesii* (Jeps.) D. P. Little (Tecate cypress) is closely related to *Callitropsis guadalupensis* [12]–[13]. The former is distributed in isolated populations associated with chaparral on the mainland from southern California to northern Baja California. We are unaware of rigorous estimates of population sizes for *C. forbesii*, but their sizes are probably smaller than those of *C. guadalupensis*. *Callitropsis forbesii* is considered in some treatments as a variety of *C. guadalupensis* [12], while in others it is treated as a separate species [13]. *Callitropsis forbesii* is classified as “subject to special protection” by the Mexican government [8].

The generic name of these taxa also is in dispute; until recently they were included in the circumscription of the genus *Cupressus*, but phylogenetic studies have shown that the genus is polyphyletic, prompting the reclassification of New World taxa (together with *Xanthocyparis* and one species of *Chamaecyparis*) under the name *Callitropsis* [13]. The name *Hesperocyparis* has also been proposed for the New World taxa [14], representing a narrower circumscription than *Callitropsis*. In this paper we adopt the name *Callitropsis*.

The reduction in size of the only population of *Callitropsis guadalupensis* provoked by grazing may have reduced its genetic diversity and negatively affected its ability to respond to future changes. An exploration of the state of its genetic diversity is a necessary condition for predicting the future prospects of the species. The main objective of this study was to characterize the genetic diversity and population structure of *C. guadalupensis* on Guadalupe Island with plastid DNA markers, which are paternally inherited in Cupressaceae [15]–[19]. By comparing genetic diversity of *C. guadalupensis* with five populations of *C. forbesii*, we observed that in contrast to general trends, the insular species (*C. guadalupensis*) maintains higher levels of genetic diversity than the continental species (*C. forbesii*). Furthermore, both species share presumably ancestral haplotypes with each other and with other New World species of *Callitropsis*. We compare the levels of DNA variation in this group with levels in other tree species, and discuss other cases of shared polymorphism among closely related species of conifers.

## Materials and Methods

For *Callitropsis guadalupensis*, five linear 200 m transects were randomly arranged in the larger of two northern forest patches and seven transects in the larger of two southern patches on Guadalupe Island. Five collections were made from trees separated by 50 m along each transect in the northern patch and one to five collections were made along the seven transects in the southern patch for a total of 54 samples. For *C. forbesii*, 20 samples were collected randomly from each of five populations from the Baja California mainland (Figure 1). The geographic location (latitude and longitude), altitude, and orientation of each population were taken with a global positioning system receiver (Table 1). The samples were placed in individual envelopes and stored at −80°C in the laboratory. Two voucher specimens for each population were deposited in the Herbario Nacional de México (MEXU).

**Table 1:** Collection data and haplotype diversity for *Callitropsis guadalupensis* and *C. forbesii*.

Location	Latitude N	Longitude W	Orientation		Altitude (m)	n	*h* ± SD
*Callitropsis guadalupensis*							
Guadalupe Island	29°06′13″	118°19′40″		Northwest	1190	54	0.698±0.053
*Callitropsis forbesii*							
Rancho El Escondido	32°33′24″	116°49′03″		North	203	20	0.537±0.104
El Dieciséis	32°28′06″	116°34′58″		South	734	20	0.189±0.108
Valle de Guadalupe	32°04′08″	116°40′39″		North	558	20	0.279±0.123
San Antonio de las Minas	31°58′13″	116°34′43″		Northeast	449	20	0.416±0.116
San Vicente	31°14′54″	116°17′11″		North	242	20	0.268±0.113

Total DNA was isolated from individual plants by grinding foliar tissue with liquid nitrogen and then using a DNeasy Plant Mini Kit (Qiagen). We evaluated 34 DNA primer pairs in a total of four individuals (two of *C. guadalupensis* and two of *C. forbesii*). Most of the primers evaluated were originally designed for *Pinus* (Pinaceae) and did not recover variation in *Callitropsis*. Only 11 primer pairs resulted in successful amplifications (Table S1) and the only markers for which we detected intraspecific variation were the *trnS*-*trnG* spacer and the *trnL*-*trnF* region (including the *trnL* intron and the *trnL*-*trnF* spacer), both of which have been used in previous intraspecific studies of Cupressaceae [19]–[20].

The *trnS*-*trnG* intergenic spacer was amplified with primers trnS^GCU^ and trnG2S [21] and the *trnL*-*trnF* region was amplified with primers Tab-C and Tab-F [22]. Polymerase chain reactions (PCR) were performed in 50 µL volumes. The final concentration of the reagents was 1X PCR buffer, 1.5 mM MgCl_2_, 200 µM of each dNTP, 1 µM of each primer, and 1 U of *Taq* polymerase. All PCR reagents were from Invitrogen. The following conditions were used for amplification of the *trnS*-*trnG* spacer: (1) 80°C for 5 min, (2) 30 cycles of 95°C for 1 min, and 55°C for 4 min (no separate extension step), and (3) 66°C for 10 min [21]. Amplification conditions for the *trnL*-*trnF* region were: (1) 94°C for 3 min, (2) 30 cycles of 94°C for 1 min, 50°C for 50 s, and 72°C for 1.2 min, and (3) 72°C for 5 min.

During early stages of the study, PCR products were purified with a Geneclean III kit (Qbiogene), diluted in double distilled water, and mailed to Macrogen, Inc. (Seoul, South Korea) for Sanger sequencing. For the remainder of the study, unpurified PCR products were mailed to the University of Washington High Throughput Genomic Unit (Seattle, Washington) for purification and sequencing. Forward and reverse sequence reads were edited and manually aligned in BioEdit Sequence Alignment Editor [23]. Sequences of each haplotype were deposited in GenBank (accessions HM147949-HM147983).

Genetic diversity was estimated for the concatenated *trnS-trnG* spacer and *trnL-trnF* region sequence alignment by comparing the number of haplotypes, adjusting the sample size to 50 individuals for each species with a rarefaction analysis [24], and calculating haplotype diversity (*h*) and nucleotide diversity (*π*)[25] with DnaSP v.4.10 [26]. To test for geographical subdivision, we compared the genetic distance for haplotypes from the same population (or species) with distances between haplotypes sampled at random using the Hudson permutation test [27].

The concatenated *trnS-trnG* spacer and *trnL-trnF* region alignment was used to estimate a genealogical network with statistical parsimony [28]. Inferred insertions or deletions (indels) were treated as single, unordered evolutionary events rather than treating them as missing data or as a fifth state. The network was constrained by assuming that base substitutions exhibited no homoplasy, but allowing homoplasy for insertions or deletions.

Phylogenetic analyses also were performed with PAUP* v.4.b10 [29]. Sequences of the *trnS-trnG* spacer and *trnL-trnF* region were obtained from single individuals representing three additional species from Mexico, *C. stephensonii* (C. B. Wolf) D. P. Little, *C. montana* (Wiggins) D. P. Little, and *C. lusitanica* (Mill.) D. P. Little. These were identical to one another and with a haplotype recovered from *C. forbesii* and *C. guadalupensis* (see below). Therefore, to include an outgroup for the phylogenetic analysis in PAUP*, sequences from the more distantly related *Juniperus monticola* Martínez were added and the matrix was realigned. The alignment was deposited in TreeBASE (http://purl.org/phylo/treebase/phylows/study/TB2:S11036). A parsimony analysis was performed both with all gaps treated as missing data and with simple indel coding [30] as implemented in SeqState v. 1.4.1 [31]. Heuristic searches were performed on the matrix with 1,000 random addition sequence replicates, tree bisection reconnection (TBR) branch swapping, and saving a maximum of 10,000 trees. A maximum likelihood analysis was also performed with gaps treated as missing characters. The best nucleotide substitution model was chosen in JModeltest [32]. Log likelihoods under different models were calculated with PHYML [33], and the Akaike Information Criterion (AIC) test was used to choose the best nucleotide substitution model. A likelihood heuristic search was performed in PAUP* with the same search parameters as the parsimony analysis. Bootstrap support was estimated from 1,000 replicates of simple sequence addition (parsimony) or as-is addition (likelihood) and TBR branch swapping. The likelihood values of inferred trees were recalculated from the non-coding portions of the plastid DNA matrix (excluding *trnS*, *trnL* and *trnF*) with and without enforcing a molecular clock and the likelihood ratio test was used to determine whether the data evolved at a homogenous rate [34].

## Results

Intra- and interspecific variation was observed in both the *trnS*-*trnG* spacer (aligned length  =  858 bps) and the *trnL-trnF* region (aligned length  =  701 bps) from 54 samples of *Callitropsis guadalupensis* and 100 samples of *C. forbesii*. Twenty-eight distinct haplotypes were detected with the *trnS-trnG* spacer, and an additional six were detected with the *trnL-trnF* region, giving a total of 34 haplotypes. For the latter marker, all variation occurred in the *trnL-trnF* spacer; the *trnL* gene and *trnL* intron were invariant. Variable sites in the *trnS-trnG* spacer included a dinucleotide microsatellite (TA) that varied from 6 to 15 repeats occurring between positions 381 and 410 of the alignment (Table S2). The microsatellite region also included three single bp indels (390, A/−; 391, T/−; 394, A/−). Outside of the microsatellite there were eight sites with substitutions (113, C/A; 191, G/A; 206, G/C; 301, C/A; 333, T/C; 440, T/C; 591, T/G; 844, T/C), a two bp indel (site 411) and a four bp indel (site 711). The variable sites in the *trnL-trnF* intergenic spacer included three single bp substitutions (334, C/T; 632, T/G, and 597, C/A), a single bp indel (site 438), a 43 bp indel (site 499), and a nine bp indel (site 544).

**Figure 2:**
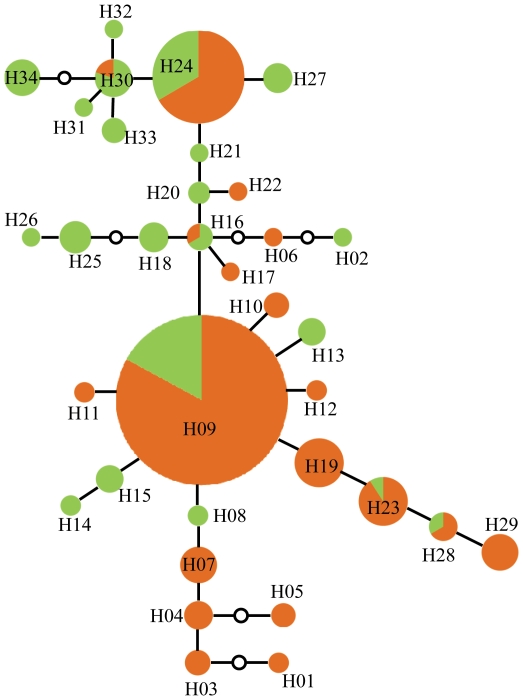
The genealogical network for *C. guadalupensis* (green) and *C. forbesii* (orange).

*Callitropsis guadalupensis* had significantly higher haplotype diversity (*h* = 0.698±0.053; mean ± s.d.) than the five populations of *C. forbesii* (*h* = 0.337±0. 054; one sample *t* test, *p*<0.001) and significantly higher nucleotide diversity (*π* = 0.00071±0.00009) compared to *C. forbesii* (*π* = 0.00024±0.00004; one sample *t* test, *p*<0.0001). Twenty-one of the 34 (62%) haplotypes were detected in *C. guadalupensis* and 19 (56%) in *C. forbesii*. The two species shared six haplotypes (H09, H16, H23, H24, H28 and H30; Figure 2). Three of the six haplotypes shared with *C. forbesii* were among those with the highest frequency in *C. guadalupensis* (H09: eight individuals, H24: six individuals, H30: four individuals); the other three haplotypes occurred at lower frequencies (H16: two individuals, H23: one individual, H28: one individual; Figure 2). Shared haplotype H09 was the most frequent in *C. forbesii*, occurring in 39 of 100 individuals, and in all five populations sampled. Thirteen haplotypes were exclusive to *C. guadalupensis* and 13 were exclusive to *C. forbesii*. This difference in the amount of genetic diversity, particularly the number of haplotypes, was tested using a rarefaction analysis in which for comparable sample sizes (50 individuals), the number of haplotypes observed in *C. guadalupensis* (20.3) was statistically greater (at the 95 and 99% confidence levels) than the number for *C. forbesii* (13.7). The Hudson, Boos and Kaplan permutation test found that genetic distances among *C. guadalupensis* haplotypes were closer than expected at random (*Snn* = 0.75081; *p* = 0.0000). Also, the permutation test within *C. forbesii* was not significant (*Snn* = 0.19205) suggesting that the taxon lacks phylogeographic structure.

Unique haplotypes were observed in four of the five populations of *C. forbesii*; only San Vicente, the most southerly population, lacked unique haplotypes. The highest haplotype and nucleotide diversity were observed in the northernmost population of *C. forbesii*, Rancho El Escondido (*h* = 0.511±0. 091; *π* = 0.00063±0.00013), and the lowest haplotype and nucleotide diversity were observed in the second northernmost population, El Dieciséis (*h* = 0.189±0. 108; *π* = 0.00022±0.00013). The Spearman rank test found no significant correlation between latitude of *C. forbesii* populations and *h* or *π* (*p*>0.05).

In the genealogical network (Figure 2), H24, which was shared with other species of *Callitropsis* (*C. lusitanica*, *C. montana*, and *C. stephensonii*), occurred at an internal node, consistent with it being ancestral. The most abundant haplotype (H09) was shared between *C. guadalupensis* and *C. forbesii* and also occurred at an internal, putatively ancestral node. Haplotypes of *C. guadalupensis* tended to occur on one side of the network and those of *C. forbesii* on the other, consistent with slow plastid DNA divergence. Also, a mismatch analysis indicated a recent population expansion for *C. forbesii* but not for *C. guadalupensis* (Fu & Li's F* and D* <0.05). This expansion was not statistically significant when Tajima's D was used for either of the species.

**Figure 3:**
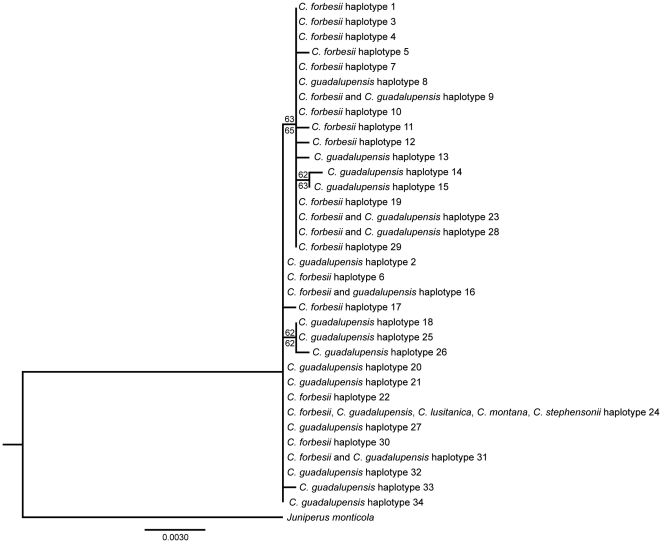
The best likelihood tree for *C. guadalupensis* and *C. forbesii*. The tree includes the 34 haplotypes from *Callitropsis* rooted with *Juniperus monticola*. The scale bar represents the estimated number of substitutions per site and the values above and below the branches are bootstrap percentages for likelihood and parsimony, respectively.

Realignment of the *trnS*-*trnG* spacer and *trnL*-*trnF* region with *Juniperus monticola* resulted in a concatenated matrix that was 1589 bp in length, with 45 variable but parsimony uninformative sites (singletons) and three parsimony informative sites. The parsimony heuristic search with gaps treated as missing recovered one equally most parsimonious tree (MPT), with a length (L) of 48 steps, a consistency index (CI) of 1.0, a consistency index excluding uninformative characters (CI_exc_) of 1.0, and a retention index (RI) of 1.0 (not shown). Using simple indel coding added 30 binary characters to the matrix, resulting in 67 variable but parsimony uninformative and 11 parsimony informative characters. The heuristic search of the matrix with simple indel coding recovered >10,000 MPTs (L = 87, CI = 0.91, CI_exc_ = 0.58, RI = 0.81). Only a single clade with two *C. guadalupensis* haplotypes (H12 and H14) was resolved in the strict consensus (not shown). For the maximum likelihood analysis, the best model using the AIC test was TPM1uf, and likelihood settings were specified as determined in JModeltest: A = 0.3435, C = 0.1458, G = 0.1590, T = 0.3517; nst = 6, rmat =  (1.000, 1.2643, 0.1849, 0.1849, 1.2643, 1.0000). The heuristic search recovered a single tree with –ln = 2383.33014 (Figure 3). In both parsimony (with gaps treated as missing) and likelihood analyses, haplotypes of *C. forbesii* and *C. guadalupensis* occurred together in an unresolved grade with zero-length branches because they differed by indels rather than substitutions. Two small clades were resolved from the majority of the haplotypes, each separated by a single substitution and recovered exclusively from *C. guadalupensis*. Haplotype 24, which was shared among *C. lusitanica*, *C. montana*, *C. stephensonii*, *C. forbesii* and *C. guadalupensis*, occurred in the unresolved grade with the majority of the *C. forbesii* and *C. guadalupensis* haplotypes. Both parsimony and likelihood bootstrap values at internal branches were low (< 70%). The log-likelihood ratio test strongly rejected the null hypothesis of rate homogeneity for the 1,453 noncoding sites (with clock −ln = 2176.66613, without clock −ln = 2284.30852, *p*<0.001).

## Discussion

### Population size, demography, and genetic diversity

Island endemics make up a disproportionate number of rare and endangered species, and although anthropogenic disturbance is a primary factor in driving species to extinction, relatively low genetic diversity of island populations is also important [2]. Consistent with this trend, another conifer, *Pinus radiata* D. Don (Pinaceae) has insular populations on both Guadalupe Island and Cedros Island in Baja California with lower levels of genetic diversity, smaller effective population sizes, and higher rates of inbreeding than disjunct continental populations in California [35]. Farther north, populations of *Pinus torreyana* Parry ex Carrière are genetically depauperate on both the mainland near San Diego and on Santa Rosa Island [36]–[37]. In contrast, *Callitropsis guadalupensis* has appreciable plastid DNA variation and shows no evidence of a founder effect or of a population bottleneck. The level of plastid DNA haplotypic diversity found in *C. guadalupensis* is similar to that found in *Cunninghamia konishii* Hayata (Cupressaceae) endemic to Taiwan (17 plastid haplotypes, *h* = 0.463) [19]. Estimates of plastid nucleotide diversity (*π*) in *C. guadalupensis* (*π* = 0.00112) are within the range of tree species in Taiwan which are hypothesized to have preserved high values of genetic diversity in one or more refugia during the Pleistocene (*Castanopsis carlesii* (Hemsl.) Hayata, *Cunninghamia konishii*, *Cyclobalanopsis glauca* Oerst., and *Trochodendron aralioides* P. F. Siebold & Zucc.; *π* = 0.00050 to 0.00200) [19], [38]. Workers have noted that species with many and/or widely distributed populations tend to have higher genetic diversity [39]–[41], but *C. guadalupensis* joins a growing list of examples of narrow endemics with relatively high genetic polymorphism [35]. Although genetic bottlenecks have been found in other components of the California offshore island biota, including the Guadalupe fur seal (*Arctocephalus townsendi*) [42] and the northern elephant seal (*Mirounga angustirostris*) [43], narrow endemic flowering plants that occur off the coast of California like *Delphinium variegatum* Torr. & A. Gray (Ranunculaceae) [44], *Castilleja grisea* Dunkle (Orobanchaceae) [45], *Jepsonia malvifolia* (Greene) Small (Saxifragaceae) [46], and *Lithophragma maximum* Bacig. (Saxifragaceae) [47], have been found to retain moderate to high genetic diversity or at least high heterozygosity despite historic grazing by goats. Other rare conifers from mainland Mexican with relatively high genetic diversity and restricted distributions include *Picea mexicana* Martínez [48] and *Pinus rzedowskii* Madrigal & M. Caball. [49].

Rare species with large local populations have shown high levels of genetic variation [50]. *Cryptomeria japonica* D. Don (Cupressaceae) includes a population on Yagu Island, Japan with relatively high isozyme diversity [51]. In this case, high diversity was attributed to the area serving as a glacial refugium and for maintaining a very large population size in a favorable climate. The estimated size of the only population of *C. guadalupensis* was 15,000 adults at the time of sampling [7], while its population 150 years ago, prior to the introduction of goats, is estimated to have been five times greater [6]. Finally, high heterozygosity has been demonstrated in species that develop in stressful environments and experience high rates of mortality of seeds and seedlings [52]. Mortality data are not available for *C. guadalupensis* or *C. forbesii*. The greater genetic variation found in *C. guadalupensis* plastid DNA compared to *C. forbesii* may be due to effectively larger historical population sizes in *C. guadalupensis*, which would suggest that it represents a stable population with a long evolutionary history [53]. The relative importance of these two factors is unknown at present, but larger historical population sizes may have been a consequence of greater environmental stability on Guadalupe Island compared to greater variation during glacial and interglacial periods on the peninsula during the Pleistocene. Although rigorous estimates are lacking for the population sizes of *C. forbesii*, the populations appear smaller than those for the only population of *C. guadalupensis*. The maximum population size of *C. guadalupensis* has probably been limited by the size of Guadalupe Island, but this still could have exceeded the population size of *C. forbesii* on the continent.

The low values of *h and π* found in *C. forbesii* are similar to other arboreal species with a wide distribution in Taiwan and East Asia, *Cyclobalanopsis glauca* [*π* = 0.00065], [54], *Trochodendron aralioides* [*π* = 0.00052], [55], and *Castanopsis carlesii* [*π* = 0.00095, 38]. The low values are consistent with the hypothesis that populations of this species recently passed through a prolonged demographic bottleneck or a founder event [53], [56]. The bottleneck hypothesis would correspond to the recent history of the species, whose wide distribution during glacial periods may have been reduced dramatically after the Pleistocene with the increase in aridity of the climate, resulting in an overall reduction into relict, mainly isolated populations [57], [58]. The more isolated southerly populations may have been the first to separate during latitudinal and altitudinal range reduction at the onset of greater aridity. A similar explanation has been offered for *Calocedrus macrolepis* Kurz (Cupressaceae, [59]). The founder effect hypothesis is consistent with the reduced variation observed in the southernmost population sampled (SV) being the result of a colonization event, possibly during the more favorable climate of the Pleistocene when the expansion of the species would have ceased. In this case, southern populations would have been peripheral and recent relative to the more ancestral northerly populations. Genetic diversity often is lower in populations that are found on the periphery or limits of distribution compared with central populations, caused by small effective population sizes, greater geographic isolation, and higher environmental pressures on the margins of distribution [60]–[63]. A combination of both hypotheses also could explain the pattern. Sampling of additional populations of *C. forbesii*, particularly those at the southern and northern limits of its distribution, would help address this question.

A complementary effect of the recent climatic history could be the increase in the frequency of fire over the previously reduced populations. *Callitropsis forbesii* is adapted to fire, which it requires for seed release and recruitment [58], [64], [65]. However, the periodicity of fire has increased to less than the 30–40 years for which it is probably adapted; this may be driving decreasing population sizes in southern California [58], [66]. Although the fire regime in Baja California seems to maintain patterns of frequency that are closer to historical levels [67], discrete events could have produced small-scale extinctions followed by recolonization of *C. forbesii*.

### Haplotype sharing across species boundaries

Of the 34 haplotypes found for *Callitropsis*, six (18%) were shared between *C. forbesii* and *C. guadalupensis*. The result of the phylogenetic analyses of the *Callitropsis* species compared in this study illustrates the extent of haplotype sharing among species and the resulting lack of reciprocal monophyly (Figures 2, 3). This could be explained by three hypotheses: 1) introgression, 2) homoplasy, or 3) incomplete lineage sorting of ancestral polymorphisms. Gene flow is unlikely because of the geographic distance between the two species (Guadalupe Island is located more than 300 km from the closest population of *C. forbesii*) and the existence of the oceanic geographic barrier. The hypothesis of homoplasy would explain the proportion of shared haplotypes due to a series of parallel and independent changes in the two species resulting in the same haplotypes arising more than once [68]. This hypothesis is unlikely considering the low rate of substitution in plastid DNA and the lack of homoplasy detected in the parsimony analysis, but inclusion of additional variable plastid DNA regions could confirm or contradict the patterns found here. The hypothesis of retention of ancestral polymorphism seems most likely and is consistent with the lack of any deep divergence between *C. forbesii* and *C. guadalupensis* plastid DNA and the discovery of the second-most frequent haplotype (H24) in three other allopatric New World species of *Callitropsis*. The most frequent haplotype overall (H09) was also shared by both species and occurred at a high frequency in all populations: Guadalupe cypress (15%) and Tecate cypress (RE: 30%, ED: 35%, VG: 40%, SA: 45% and SV: 60%). The persistence of ancestral haplotypes could be associated with the climatic or ecological conditions of the habitats where it occurs (i.e., they could be favored by selection), but it is more likely that they are the result of slow coalescence favored by large effective population sizes, long generation times, and a low mutation rate.

The silent substitution rate of plastid DNA is estimated at 0.22–0.42×10^−9^ substitutions/site/year in the conifer family Pinaceae [69] compared to a rate of 1.0–3.0×10^−9^ estimated in angiosperms [70]. The lower *Pinus* rate is probably closer to the rate for *Callitropsis* because the two genera have similarly long life spans and large population sizes and because of their relative phylogenetic proximity. Although the substitution rate of the *trnS-G* and *trnL-F* spacers deviated significantly from a molecular clock, a single substitution in 1453 bps (excluding the *trn* genes) would be expected to occur every ∼ 1,600,000 to 3,100,000 years. *Callitropsis* haplotypes differ by zero to four substitutions. Two clades with haplotypes exclusive to Guadalupe Island (H14 and H15, and H18, H25, and H26) are separated from the remaining haplotypes by a single substitution, suggesting that they originated subsequent to the formation of the island approximately 7 million years ago [4]. Next generation sequencing of plastid genomes should be able to provide more data, and in turn more accurate measures of sequence divergence and of plastid DNA ages in *Callitropsis* [37].

Non-monophyly of plastid DNA may be a common pattern in recently diverged conifer species pairs and in species complexes. It has been documented in other species rich conifer genera such as *Pinus* [71]–[73], *Abies* [74], and *Juniperus* [75]. Interspecific gene flow has been implicated as causing non-monophyly of plastid DNA in *Pinus lambertiana* Douglas [72], but the relative contribution of retention of ancestral polymorphism versus hybridization and introgression are less clear in other cases.

### Conservation implications

Seed collection and in situ and ex situ conservation need to be sustained, and should continue to distinguish between seed collected from *C. guadalupensis* and *C. forbesii*. In the case of *C. forbesii*, genetic diversity is highest in two of the three most northerly populations (Rancho El Escondido and Valle de Guadalupe). The greatest number of plastid haplotypes would be preserved if conservation efforts focused on northern populations, but studies of more populations using higher resolution multi-locus markers are needed.

## Supporting Information

Table S1 Plastid primer pairs that gave successful PCR reactions in *Callitropsis*.
(DOC)

Table S2 Variable sites and their position in the alignments of the *trnS-trnG* intergenic spacer and the *trnL-trnF* region.
(DOC)
