# Supplementary material for: Plastid DNA Diversity Is Higher in the Island Endemic Guadalupe Cypress than in the Continental Tecate Cypress
Source: PLoS One. 2011 Jan 20;6(1):e16133. doi: 10.1371/journal.pone.0016133 (PMC3024418; doi:10.1371/journal.pone.0016133)
Supplement: Table S1 — Plastid primer pairs that gave successful PCR reactions in Callitropsis. (DOC) [file pone.0016133.s001.doc]

Table S1. Plastid primer pairs that gave successful PCR reactions in *Callitropsis*.

| **Description** | **Sequence (5'-3')** | **Position in *Pinus thunbergii*** | **Length (bp)** | **Reference** |
| --- | --- | --- | --- | --- |
| *trn*S, *psb*Z | GAG AGA GAG GGA TTC GAA CC | 7894 | 327 | [21] |
|  | CGA TTG GAA CGC AAT AGT CA | - |  | This study |
| *trn*S-*trn*G | AGA TAG GGA TTC GAA CCC TCG GT | 7919 | 848 | [21] |
|  | TTT TAC CAC TAA ACT ATA CCC GC | 8845 |  | [21] |
| *trn*G intron | GCG GGT ATA GTT TAG TGG TAA AA | 8867 | 790 | [21] |
|  | GTA GCG GGA ATC GAA CCC GCA TC | 9629 |  | [21] |
| *trn*D-*trn*Y-*trn*E | CAG GGC GGT ACT CTA ACC AA | 28456 | 384 | This study |
|  | TCT CTT TCA AGG AGG CAA CG | 28893 |  | This study |
| *pet*B partial*,* groupIII intron*, orf*52 | GGA TGG GAT TCC GGT TAG TT | 54663 | N/S | This study |
|  | TCA ATC CAA ACC TCA GAT TTC A | 55216 |  | This study |
| *trn*L-*trn*F | ATT TGA ACT GGT GAC ACG AG | 67757 | 682 | [22] |
|  | CGA AAT CGG TAG ACG CTA CG | 68720 |  | [22] |
| *trn*T-*trn*L | TCT ACC GAT TTC GCC ATA TC | 68746 | 394 | [22] |
|  | CAT TAC AAA TGC GAT GCT CT | 69195 |  | [22] |
| *trn*S-*trn*G | GAT CGA ACC CGC ATC TTC T | 79135 | 713 | This study |
|  | GGG ATT CGA ACC CTC GAT A | 79939 |  | This study |
| *orf*42d, 77, 42e, | TCG TCT TGA ACC TTC TGC AA | 82936 | N/S | This study |
|  | TCT GGT ATT GAA ATT TGA TGG AGA | 83475 |  | This study |
| *ycf1* | GAC GGA AAA TTA TGA TAT GGA AGA | 97759 | N/S | This study |
|  | CAA GGC TTC AAA CGA AAA GG | 98212 |  | This study |
| *ycf6* | ATG GAT ATA GTA AGT CTY GCT TGG GC | - | N/S | [21] |
|  | TCT CTG GAA AAA GGA ACT TTA TGC | - |  | This study |
