# Supplementary material for: Plastid DNA Diversity Is Higher in the Island Endemic Guadalupe Cypress than in the Continental Tecate Cypress
Source: PLoS One. 2011 Jan 20;6(1):e16133. doi: 10.1371/journal.pone.0016133 (PMC3024418; doi:10.1371/journal.pone.0016133)
Supplement: Table S2 — Variable sites and their position in the alignments of the trnS-trnG intergenic spacer and the trnL-trnF region. (DOC) [file pone.0016133.s002.doc]

**Table S2.** Variable sites and their position in the alignments of the *trnS-trnG* intergenic spacer and the *trnL-trnF* region.

|  | ***trnS-trnG*** | | | | | | | | | | | | ***trnL-trnF*** | | | | | |  |  |
| --- | --- | --- | --- | --- | --- | --- | --- | --- | --- | --- | --- | --- | --- | --- | --- | --- | --- | --- | --- | --- |
| **Haplotype** | **113** | **191** | **206** | **301** | **333** | **381 - 410** | | **411** | **440** | **591** | **711** | **844** | **4**  **34** | **438** | **499 - 541** | **544 - 552** | **597** | **632** | **n** | **Species*** |
| H01 | A | G | G | C | T | TATATATATATATATATATATATATATATA | ─ | | T | T | ─ | T | C | G | ─ | § | C | T | 1 | *Cf* |
| H02 | C | . | . | . | . | TATATATATATATATATATATATATATATA | . | | . | . | . | . | . | . | . | . | . | . | 1 | *Cg* |
| H03 | . | . | . | . | . | TATATATATA─ATATATATATATATATA | . | | . | . | . | . | . | . | . | . | . | . | 2 | *Cf* |
| H04 | . | . | . | . | . | TATATATATATATATATATATATATA | . | | . | . | . | . | . | . | . | . | . | . | 3 | *Cf* |
| H05 | . | A | . | . | . | TATATATATATATATATATATATATA | . | | . | . | † | . | . | . | . | . | . | . | 1 | *Cf* |
| H06 | C | . | . | . | . | TATATATATATATATATATATATATA | . | | . | . | . | . | . | . | . | . | . | . | 1 | *Cf* |
| H07 | . | . | . | . | . | TATATATATATATATATATATATA | . | | . | . | . | . | . | . | . | . | . | . | 5 | *Cf* |
| H08 | . | . | . | . | . | TATATATATA─ATATATATATATA | . | | . | . | . | . | . | . | . | . | . | . | 1 | *Cg* |
| H09 | . | . | . | . | . | TATATATATATATATATATATA | . | | . | . | . | . | . | . | . | . | . | . | 47 | *Cf*(39), *Cg*(8) |
| H10 | . | . | . | . | . | TATATATATATATATATATATA | . | | . | . | . | . | . | ─ | . | . | . | . | 3 | *Cf* |
| H11 | . | . | . | . | . | TATATATATATATATATATATA | . | | . | G | . | . | . | . | . | . | . | . | 1 | *Cf* |
| H12 | . | . | T | . | . | TATATATATATATATATATATA | . | | . | . | . | . | . | . | . | . | . | . | 1 | *Cf* |
| H13 | . | . | . | . | . | TATATATATATATATATATATA | . | | . | . | . | C | . | . | . | . | . | . | 3 | *Cg* |
| H14 | . | . | . | A | C | TATATATATATATATATATATA | . | | . | . | . | . | . | . | . | . | . | . | 1 | *Cg* |
| H15 | . | . | . | . | C | TATATATATATATATATATATA | . | | . | . | . | . | . | . | . | . | . | . | 2 | *Cg* |
| H16 | C | . | . | . | . | TATATATATATATATATATATA | . | | . | . | . | . | . | . | . | . | . | . | 3 | *Cf*(1), *Cg*(2) |
| H17 | C | . | . | . | . | TATATATATATATATATATATA | . | | . | . | . | . | . | . | . | . | A | . | 1 | *Cf* |
| H18 | C | . | . | . | . | TATATATATATATATATATATA | . | | C | . | . | . | . | . | . | . | . | . | 3 | *Cg* |
| H19 | . | . | . | . | . | TATATATATATATATATATA | . | | . | . | . | . | . | . | . | . | . | . | 10 | *Cf* |
| H20 | C | . | . | . | . | TATATATATATATATATATA | . | | . | . | . | . | . | . | . | . | . | . | 2 | *Cg* |
| H21 | C | . | . | . | . | TATATATAT─TATATATATA | . | | . | . | . | . | . | . | . | . | . | . | 1 | *Cg* |
| H22 | C | . | . | . | . | TATATATATATAT─TATATA | . | | . | . | . | . | . | . | . | . | . | . | 1 | *Cf* |
| H23 | . | . | . | . | . | TATATATATATATATATA | . | | . | . | . | . | . | . | . | . | . | . | 11 | *Cf* (10), *Cg*(1) |
| H24 | C | . | . | . | . | TATATATATATATATATA | . | | . | . | . | . | . | . | . | . | . | . | 18 | *Cf* (12), *Cg*(6) |
| H25 | C | . | . | . | . | TATATATATATATATATA | . | | C | . | . | . | . | . | . | . | . | . | 4 | *Cg* |
| H26 | C | . | . | . | . | TATATATATATATATATA | . | | C | . | . | . | T | . | . | . | . | . | 1 | *Cg* |
| H27 | C | . | . | . | . | TATATATATATATATATA | TT | | . | . | . | . | . | . | . | . | . | . | 3 | *Cg* |
| H28 | . | . | . | . | . | TATATATATATATATA | . | | . | . | . | . | . | . | . | . | . | . | 3 | *Cf* (2), *Cg*(1) |
| H29 | . | . | . | . | . | TATATATATATATATA | TT | | . | . | . | . | . | . | . | . | . | . | 5 | *Cf* |
| H30 | C | . | . | . | . | TATATATATATATATA | . | | . | . | . | . | . | . | . | . | . | . | 5 | *Cf* (1), *Cg*(4) |
| H31 | C | . | . | . | . | TATATATATATATATA | . | | . | . | . | . | . | . | ‡ | . | . | . | 1 | *Cg* |
| H32 | C | . | . | . | . | TATATATATATATATA | . | | . | . | . | . | . | . | . | ─ | . | . | 1 | *Cg* |
| H33 | C | . | . | . | . | TATATATATATATATA | . | | . | . | . | . | . | . | . | . | . | G | 2 | *Cg* |
| H34 | C | . | . | . | . | TATATATATATA | . | | . | . | . | . |  | . | . | . | . |  | 6 | *Cg* |

*, *Cf* = *Callitropsis forbesii* and *Cg* = *Callitropsis guadalupensis*: †, AAAT; ‡,TACAATTAAATAATACACAATATATAATATTGTGTATTATTTA; §, TGTATAAAT
